# Supplementary material for: Proteogenomic analysis reveals adaptive strategies for alleviating the consequences of aneuploidy in cancer
Source: EMBO J. 2025 Feb 10;44(6):1829–65. doi: 10.1038/s44318-025-00372-w (PMC11914506; doi:10.1038/s44318-025-00372-w)
Supplement: Supplementary file 1 — Appendix [file 44318_2025_372_MOESM1_ESM.pdf]

Appendix

Proteogenomic analysis reveals adaptive strategies to alleviate the consequences of aneuploidy in cancer

Jan-Eric Bökenkamp[1, #], Kristina Keuper[1,2, #], Stefan Redel[1], Karen Barthel[1], Leah Johnson[1], Amelie Becker[1], Angela Wieland[1], Markus Raeschle[1], Zuzana Storchova[1],*

1 RPTU Kaiserslautern-Landau, Paul- Ehrlich Strasse 24, 67663 Kaiserslautern, Germany

2 Danish Cancer Institute, Strandboulevarden 49, 2100 Copenhagen, Denmark

* Corresponding author: Zuzana Storchova, storchova@bio.uni-kl.de

[#] Equal contribution

Table of Contents

Appendix Figure S1 3
Appendix Figure S2 5
Appendix Figure S3 7
Appendix Figure S4 9
Appendix Figure S5 11
Appendix Figure S6 13
Appendix Figure S7 15
Appendix Figure S8 17
Appendix Figure S9 19

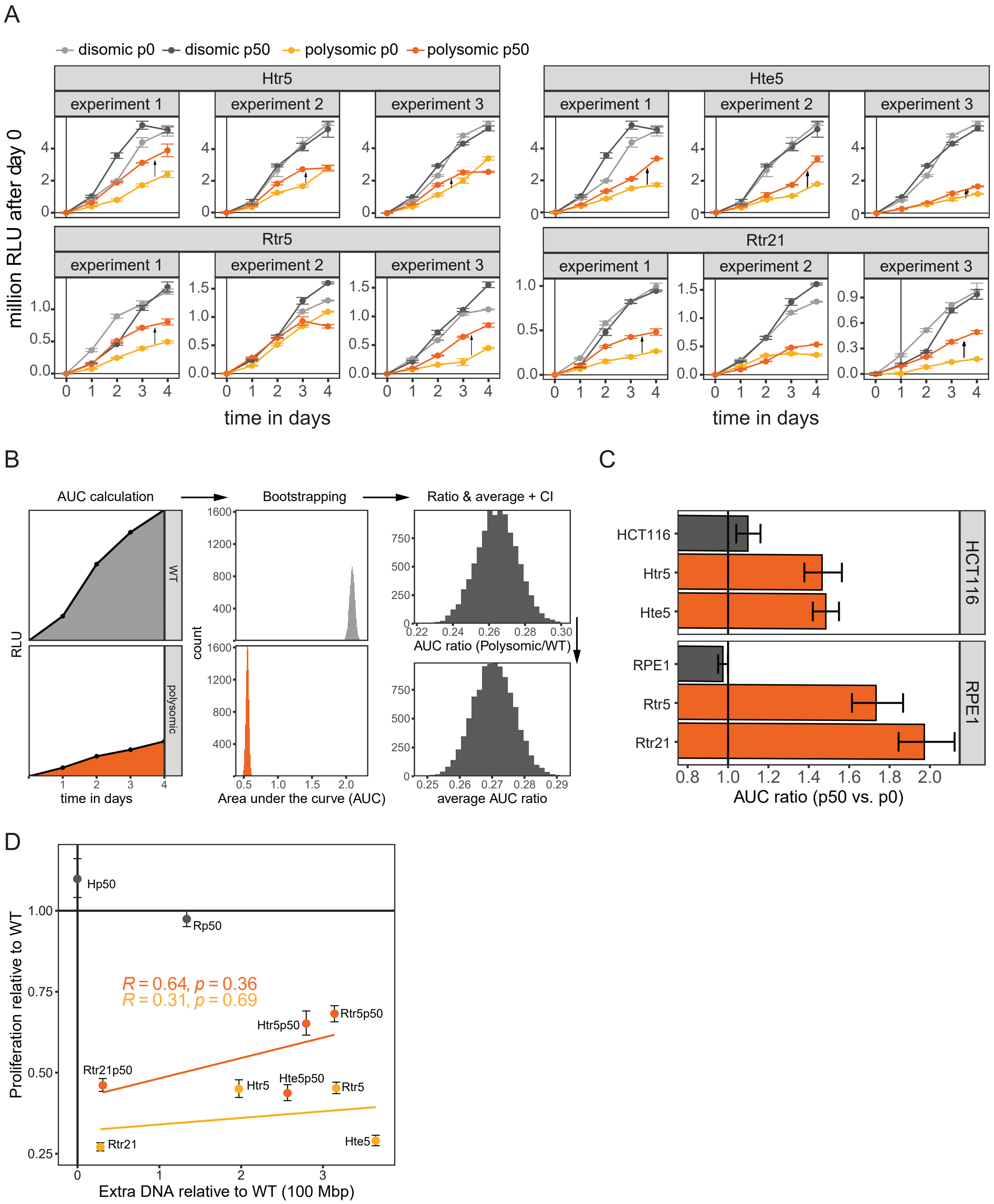
A
disomic p0
disomic p50
polysomic p0
polysomic p50
Htr5
Hte5
Rtr5
Rtr21
experiment 1
experiment 2
experiment 3
million RLU after day 0
time in days
B
AUC calculation
Bootstrapping
Ratio & average + CI
RLU
WT
polysomic
time in days
count
Area under the curve (AUC)
AUC ratio (Polysomic/WT)
average AUC ratio
C
HCT116
Htr5
Hte5
RPE1
Rtr5
Rtr21
AUC ratio (p50 vs. p0)
D
Hp50
Rp50
R = 0.64, p = 0.36
R = 0.31, p = 0.69
Htr5p50
Rtr5p50
Rtr21p50
Htr5
Hte5p50
Rtr5
Rtr21
Hte5
Proliferation relative to WT
Extra DNA relative to WT (100 Mbp)

Appendix Figure S1 - Evaluation of the proliferation changes in the polysomic cells after in vitro evolution

A. Growth curves from all experiments of population growth of the cell lines before and after *in vitro* evolution evaluated by MTT assay and normalized to the time point 0. Points represent mean relative light units (RLU) of 2 to 3 seeded replicates. Error bars represent SEM.

B. Schematic of growth curve analysis pipeline consisting of calculating, bootstrapping, normalizing and averaging the areas under the curve (AUC) per experiment (n = 3).

C. Ratios of AUCs between passage 50 and passage 0 cell lines. Error bars represent empirical 95% confidence internals (10000 bootstrap samples).

D. Correlation of the change in proliferation and change in total DNA relative to unevolved WT. Values depict Pearson correlation coefficient and p-value for p0 and p50 polysomic cell lines, error bars represent empirical 95% confidence intervals.

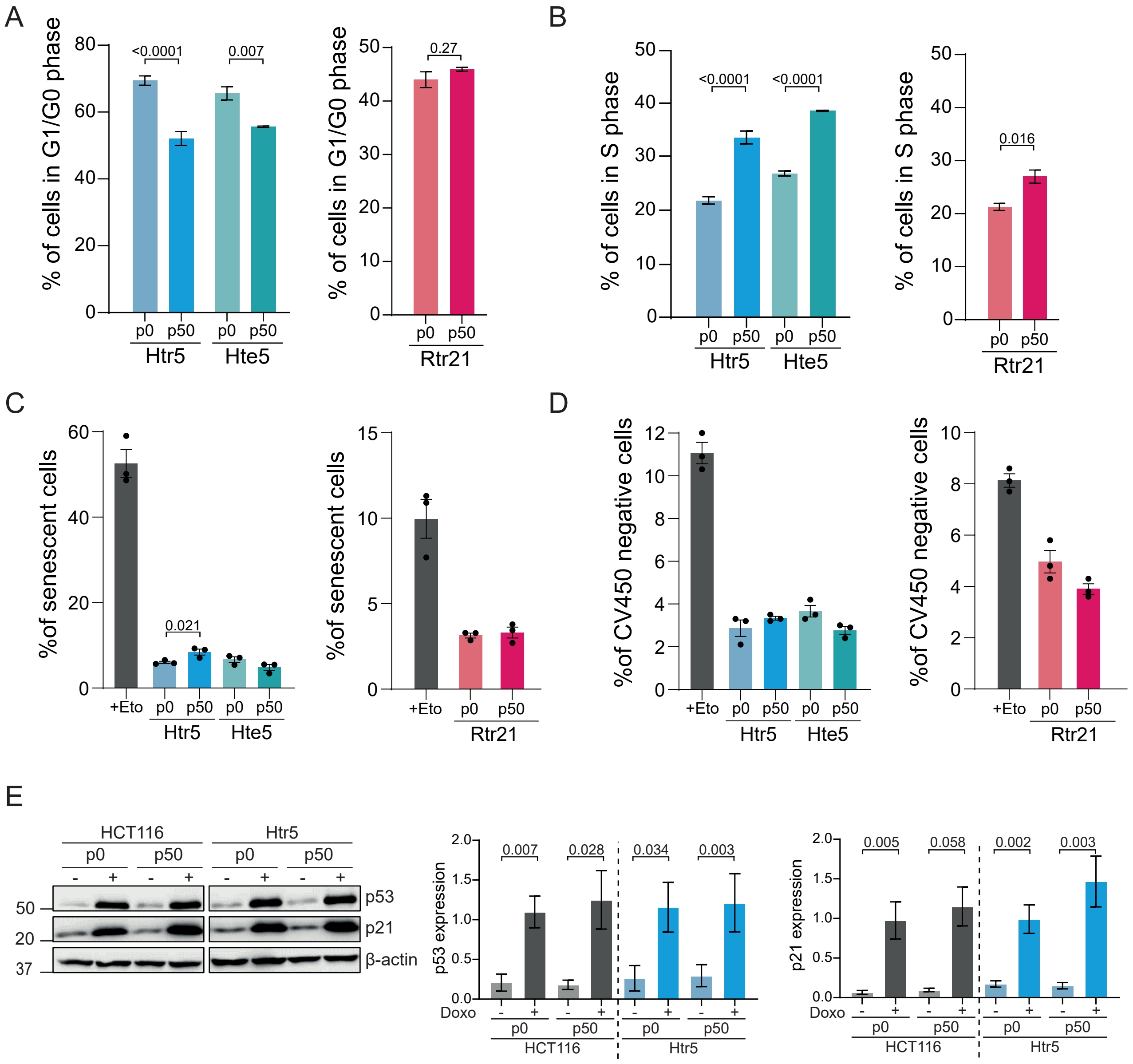
A
% of cells in G1/G0 phase
<0.0001
0.007
p0 p50 p0 p50
Htr5 Hte5
0.27
p0 p50
Rtr21
B
% of cells in S phase
<0.0001
<0.0001
p0 p50 p0 p50
Htr5 Hte5
0.016
p0 p50
Rtr21
C
%of senescent cells
0.021
+Eto p0 p50 p0 p50
Htr5 Hte5
+Eto p0 p50
Rtr21
D
%of CV450 negative cells
+Eto p0 p50 p0 p50
Htr5 Hte5
+Eto p0 p50
Rtr21
E
HCT116 Htr5
p0 p50 p0 p50
50 20 37
p53
p21
β-actin
p53 expression
0.007 0.028 0.034 0.003
p21 expression
0.005 0.058 0.002 0.003
Doxo
p0 p50 p0 p50
HCT116 Htr5

Appendix Figure S2 - Evaluation of the cell cycle, cell death, senescence and p53 signaling in the polysomic cells after *in vitro* evolution

A. Relative proportion of cells in G1/G0 phase in polysomic cell populations before and after *in vitro* evolution determined with flow cytometry using EdU incorporation and DAPI staining (n: 3 - 9).

B. Relative proportion of cells in S phase in polysomic cells before and after evolution determined with flow cytometry using EdU incorporation and DAPI staining (n: 3 - 9).

C. Senescence in polysomic cells before and after *in vitro* evolution (n = 3).

D. Proportion of CV450 viability dye negative cells in polysomic cell populations before and after *in vitro* evolution (n = 3).

E. Representative immunoblot of p53 and p21 before and after *in vitro* evolution with and without doxorubicin treatment and quantifications. Doxorubicin treatment was performed with 1 µM overnight (n: 4 - 5).

F. Representative image and quantification of γH2Ax immunofluorescence before and after *in vitro* evolution in polysomic cells. Three biological replicates; At least 5000 nuclei were scored with each sample. Scale bars in microscopy images: 10 µm.

Data information: Bar plots show mean with SEM is shown in all bar plots. P-values were calculated using unpaired Student's t-test.

A

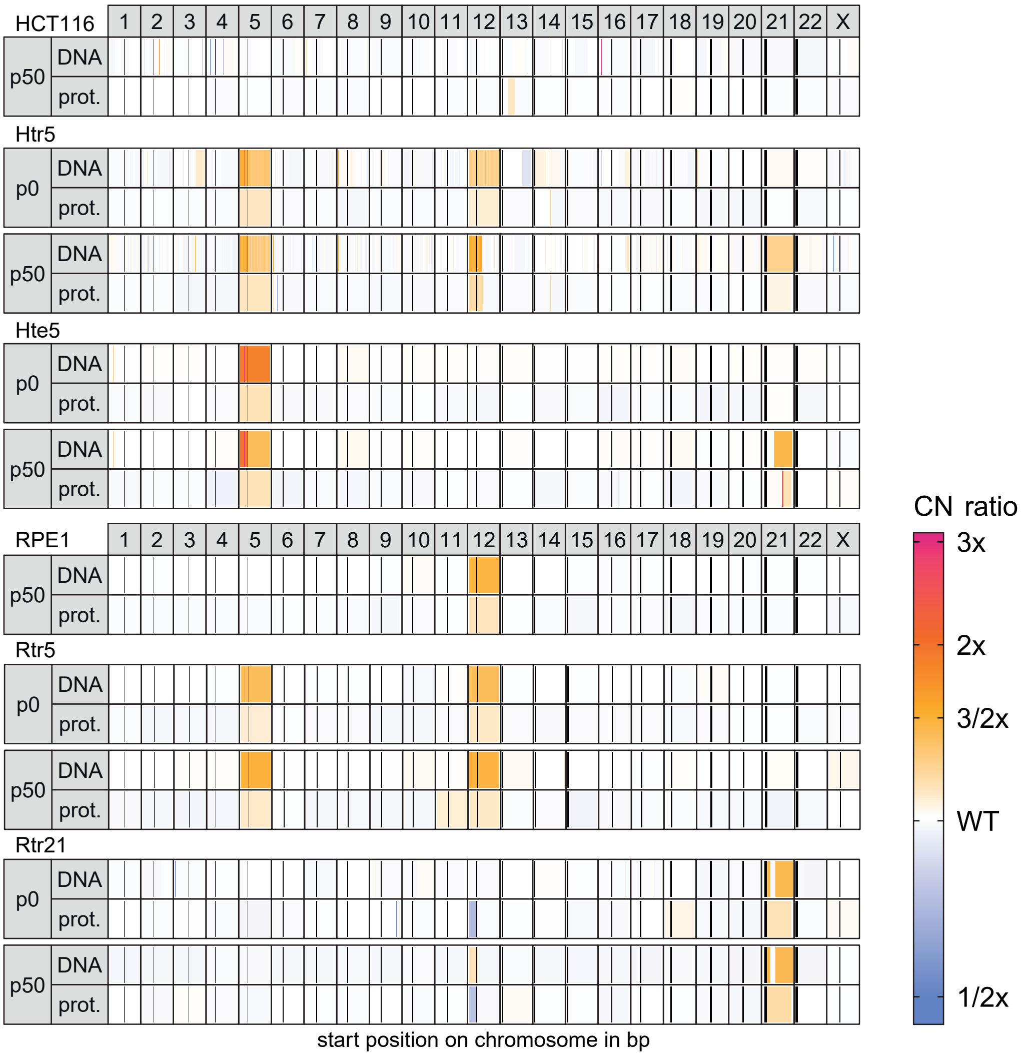
HCT116
1 2 3 4 5 6 7 8 9 10 11 12 13 14 15 16 17 18 19 20 21 22 X
p50
DNA
prot.
Htr5
p0
p50
Hte5
RPE1
Rtr5
Rtr21
start position on chromosome in bp
CN ratio
3x
2x
3/2x
WT
1/2x

Appendix Figure S3 - Genome and proteome changes before and after *in vitro* evolution

A. DNA copy number ratios and protein abundance fold changes relative to parental, unevolved WT for each cell line (row) and chromosome (column) as grouped by circular binary segmentation. Vertical bars within chromosomes depict the location of centromeres.

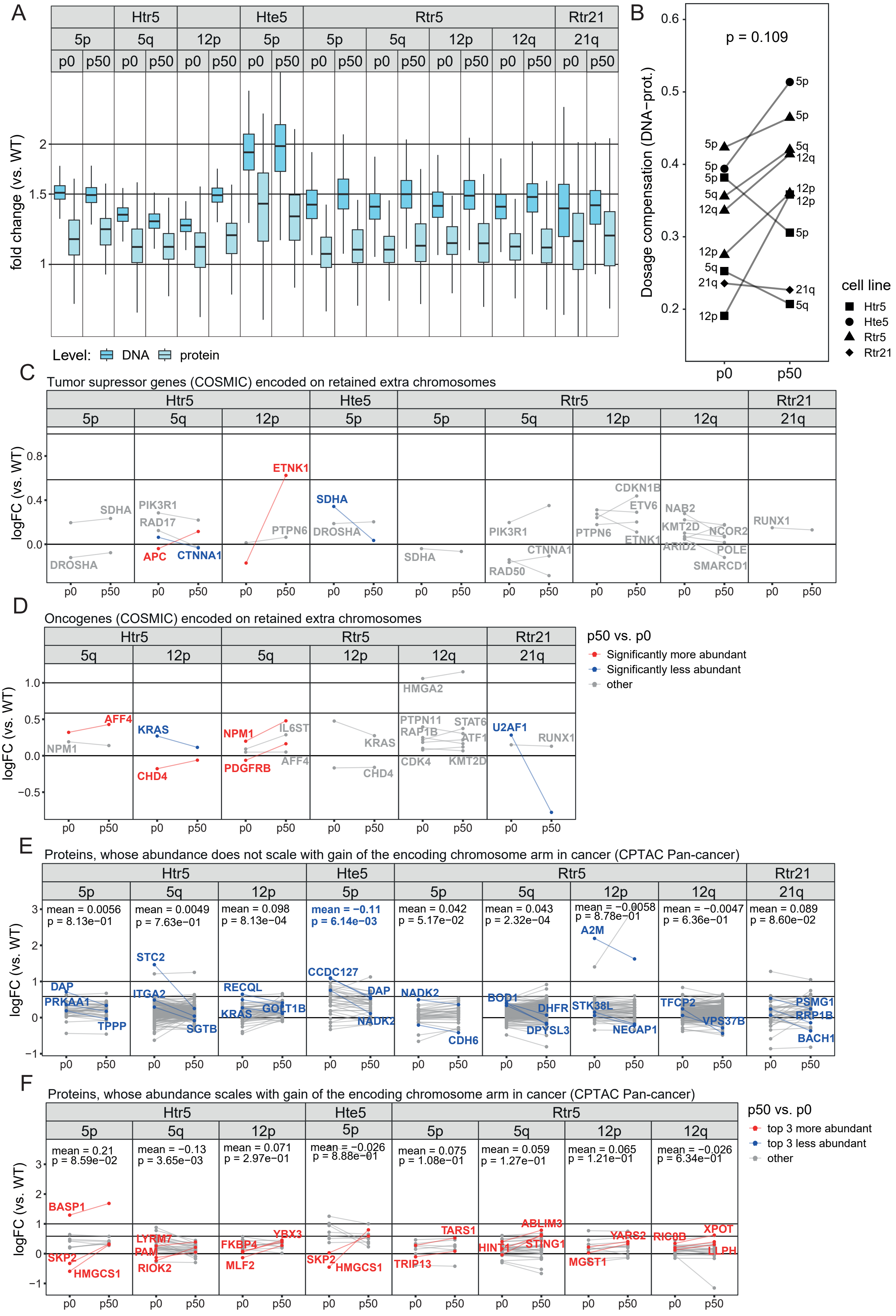
A
Htr5
Hte5
Rtr5
Rtr21
5p
5q
12p
12q
21q
p0
p50
fold change (vs. WT)
2
1.5
1
Level:
DNA
protein
B
p = 0.109
Dosage compensation (DNA−prot.)
0.6
0.5
0.4
0.3
0.2
cell line
Htr5
Hte5
Rtr5
Rtr21
C
Tumor supressor genes (COSMIC) encoded on retained extra chromosomes
logFC (vs. WT)
SDHA
DROSHA
PIK3R1
RAD17
APC
CTNNA1
ETNK1
PTPN6
RAD50
CDKN1B
ETV6
NAB2
KMT2D
NCOR2
ARID2
POLE
SMARCD1
RUNX1
D
Oncogenes (COSMIC) encoded on retained extra chromosomes
AFF4
NPM1
KRAS
CHD4
PDGFRB
IL6ST
HMGA2
PTPN11
STAT6
RAP1B
ATF1
CDK4
KMT2D
U2AF1
RUNX1
p50 vs. p0
Significantly more abundant
Significantly less abundant
other
E
Proteins, whose abundance does not scale with gain of the encoding chromosome arm in cancer (CPTAC Pan-cancer)
mean = 0.0056
p = 8.13e−01
mean = 0.0049
p = 7.63e−01
mean = 0.098
p = 8.13e−04
mean = −0.11
p = 6.14e−03
mean = 0.042
p = 5.17e−02
mean = 0.043
p = 2.32e−04
mean = −0.0058
p = 8.78e−01
mean = −0.0047
p = 6.36e−01
mean = 0.089
p = 8.60e−02
DAP
PRKAA1
TPPP
STC2
ITGA2
SGTB
RECQL
KRAS
GOLT1B
CCDC127
DAP
NADK2
NADK2
CDH6
BOD1
DHFR
DPYSL3
A2M
STK38L
NECAP1
TFCP2
VPS37B
PSMG1
RRP1B
BACH1
F
Proteins, whose abundance scales with gain of the encoding chromosome arm in cancer (CPTAC Pan-cancer)
mean = 0.21
p = 8.59e−02
mean = −0.13
p = 3.65e−03
mean = 0.071
p = 2.97e−01
mean = −0.026
p = 8.88e−01
mean = 0.075
p = 1.08e−01
mean = 0.059
p = 1.27e−01
mean = 0.065
p = 1.21e−01
mean = −0.026
p = 6.34e−01
BASP1
SKP2
HMGCS1
LYRM7
PAM
RIOK2
FKBP4
YBX3
MLF2
SKP2
HMGCS1
TARS1
TRIP13
ABLIM3
HINT1
STING1
YARS2
MGST1
RIC8B
XPOT
LLPH
p50 vs. p0
top 3 more abundant
top 3 less abundant
other

Appendix Figure S4 – Dosage compensation of proteins before and after *in vitro* evolution

A. Fold change in DNA copy numbers and protein abundances relative to parental, unevolved WT for supernumerary chromosome arms, which were not lost during *in vitro* evolution.

B. Chromosome arm-level dosage compensation, calculated as the difference between average DNA copy number and protein abundance fold changes, before (p0) and after (p50) *in vitro* evolution.

C. Average log2 protein dosage changes relative to disomic parent before (p0) and after (p50) *in vitro* evolution of Tier 1 and Tier 2 tumor suppressor genes from the Cancer Gene Consensus of the COSMIC database.

D. Average log2 protein dosage changes relative to disomic parent before (p0) and after (p50) *in vitro* evolution of Tier 1 and Tier 2 oncogenes from the Cancer Gene Consensus of the COSMIC database.

E. Average log2 protein dosage changes relative to disomic parent before (p0) and after (p50) *in vitro* evolution of proteins whose abundance does not scale with the gain of the encoding chromosome arm in CPTAC tumors (See Methods).

F. Average log2 protein dosage changes relative to disomic parent before (p0) and after (p50) *in vitro* evolution of proteins whose abundance scales with the gain of the encoding chromosome arm in CPTAC tumors (See Methods).

Data information: Mean differences in dosage compensation and protein dosage changes were tested for statistical significance using paired Student's t-tests.

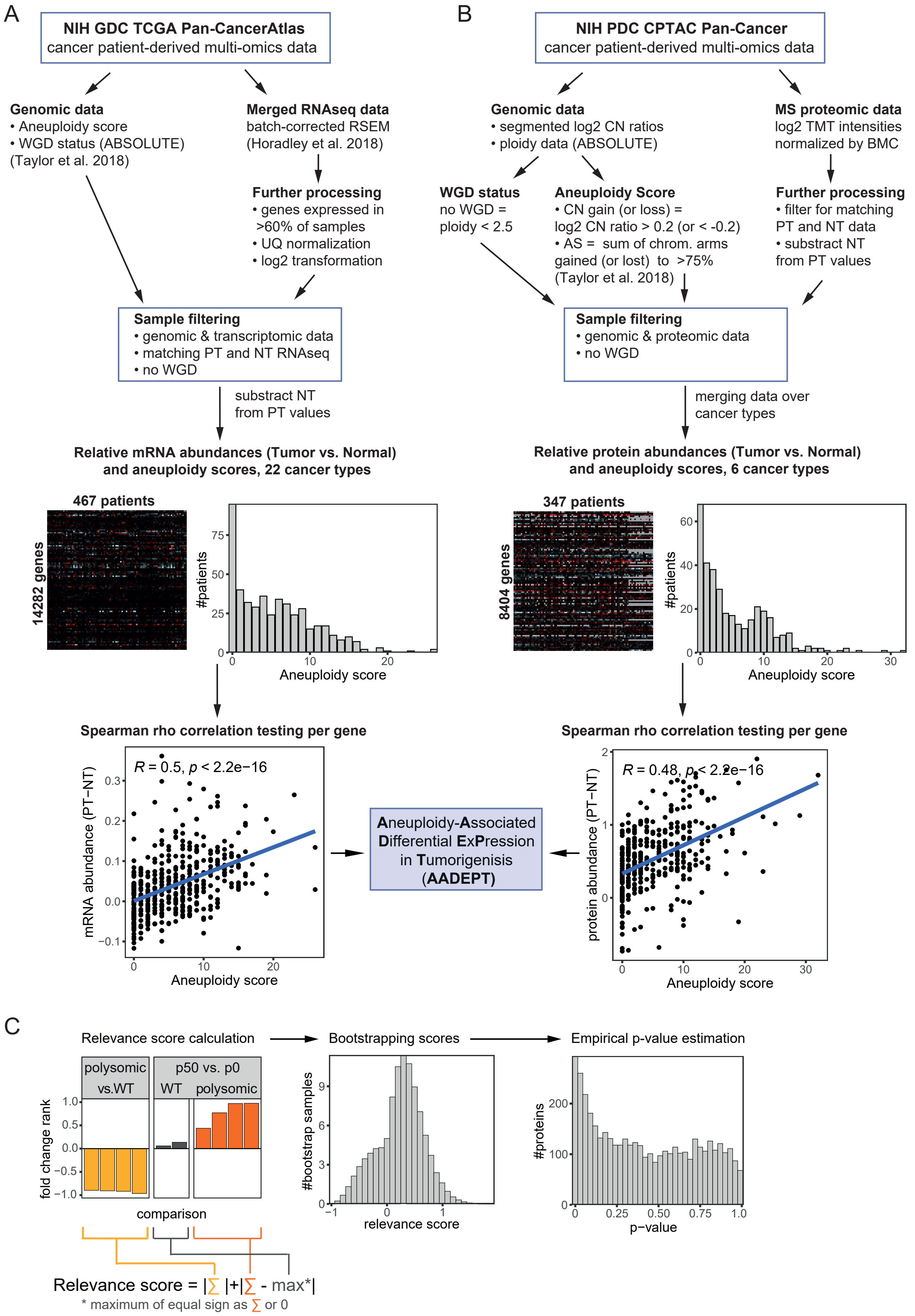
A
NIH GDC TCGA Pan-CancerAtlas
cancer patient-derived multi-omics data
Genomic data
• Aneuploidy score
• WGD status (ABSOLUTE)
(Taylor et al. 2018)
Merged RNAseq data
batch-corrected RSEM
(Horadley et al. 2018)
Further processing
• genes expressed in >60% of samples
• UQ normalization
• log2 transformation
Sample filtering
• genomic & transcriptomic data
• matching PT and NT RNAseq
• no WGD
substract NT from PT values
Relative mRNA abundances (Tumor vs. Normal) and aneuploidy scores, 22 cancer types
467 patients
14282 genes
#patients
Aneuploidy score
Spearman rho correlation testing per gene
R = 0.5, p < 2.2e−16
mRNA abundance (PT−NT)
Aneuploidy score
B
NIH PDC CPTAC Pan-Cancer
cancer patient-derived multi-omics data
Genomic data
• segmented log2 CN ratios
• ploidy data (ABSOLUTE)
MS proteomic data
log2 TMT intensities
normalized by BMC
WGD status
no WGD = ploidy < 2.5
Aneuploidy Score
• CN gain (or loss) = log2 CN ratio > 0.2 (or < -0.2)
• AS = sum of chrom. arms gained (or lost) to >75%
(Taylor et al. 2018)
Further processing
• filter for matching PT and NT data
• substract NT from PT values
Sample filtering
• genomic & proteomic data
• no WGD
merging data over cancer types
Relative protein abundances (Tumor vs. Normal) and aneuploidy scores, 6 cancer types
347 patients
8404 genes
#patients
Aneuploidy score
Spearman rho correlation testing per gene
R = 0.48, p < 2.2e−16
protein abundance (PT−NT)
Aneuploidy score
Aneuploidy-Associated Differential ExPression in Tumorigenisis (AADEPT)
C
Relevance score calculation
Bootstrapping scores
Empirical p-value estimation
polysomic vs.WT
p50 vs. p0
WT
polysomic
fold change rank
comparison
#bootstrap samples
relevance score
#proteins
p−value
Relevance score = |∑ |+|∑ - max*|
* maximum of equal sign as ∑ or 0

Appendix Figure S5 - Schematic depiction of the strategy to calculate AADEPT scores and the aneuploidy relevance score

A. Derivation of AADEPT scores as Spearman correlation coefficients between the degree in aneuploidy and the change in gene expression in cancer patients (primary tumor vs. normal tissue) from multi-omics data of TCGA Pan-Cancer Atlas and

B. from the Clinical Proteomic Tumor Analysis Consortium.

C. Scoring of proteins based on relevant expression change patterns in our evolving polysomic model cell lines and deriving empirical p-values by bootstrapping the calculated scores. The relevance score is defined as the absolute sum of protein abundance fold change ranks (scaled from -1 to 1) of comparisons between evolved and unevolved polysomic cell lines as well as unevolved polysomic cell lines and their respective wild type. Congruent changes in wild type cells with *in vitro* evolution are penalized by subtracting the maximum fold change rank of equal sign.

Data information: GDC = Genomic Data Commons, PDC = Proteomic Data Commons, TCGA = The Cancer Genome Atlas, CPTAC = Clinical Proteomic Tumor Analysis Consortium, BMC = Baylor College of Medicine, AS = aneuploidy score, WGD = whole-genome doubling, PT = primary tumor, NT = normal tissue, CN = copy number, UQ = upper-quartile.

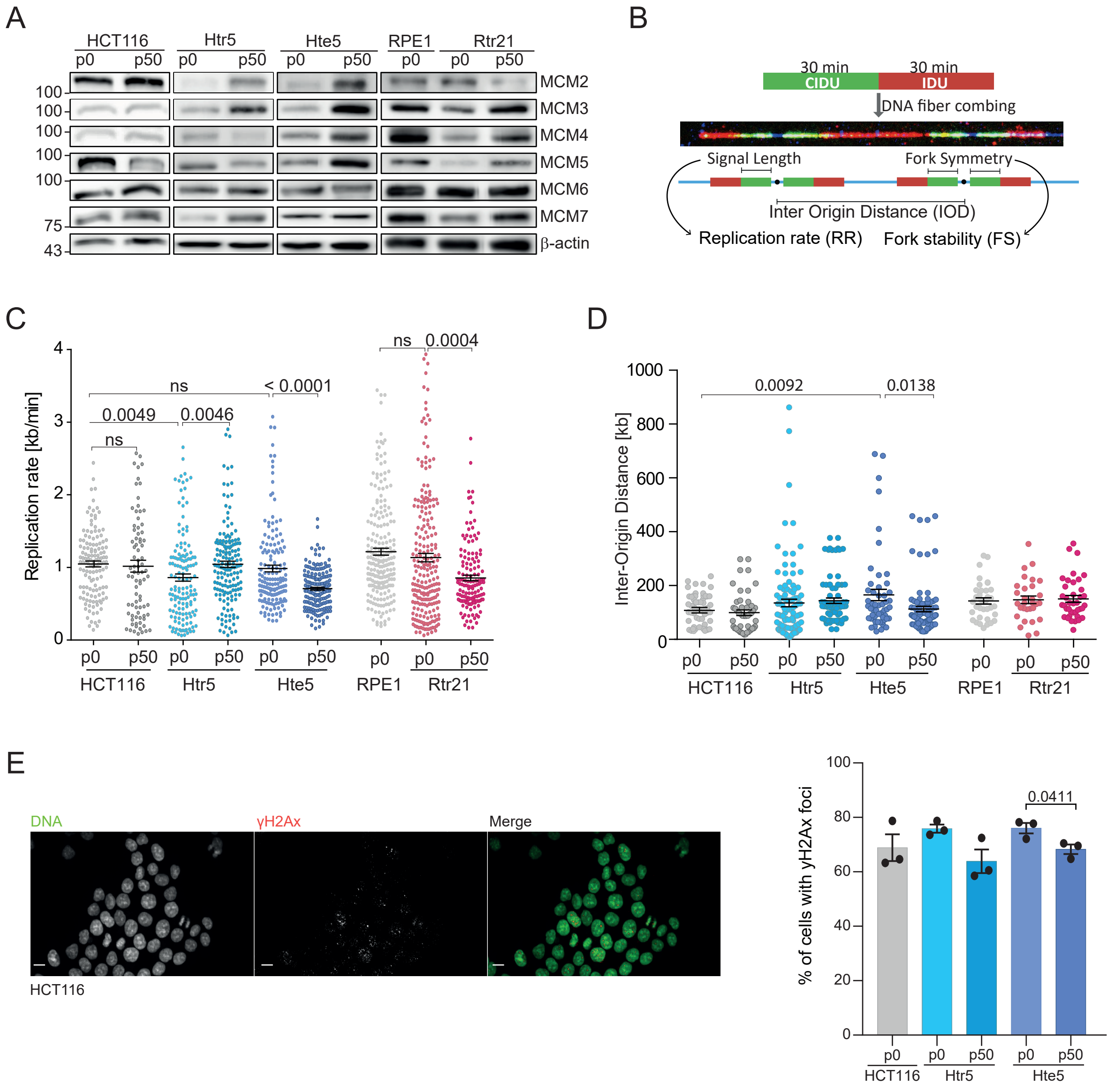
A
HCT116
Htr5
Hte5
RPE1
Rtr21
p0
p50
MCM2
MCM3
MCM4
MCM5
MCM6
MCM7
β-actin
100
75
43
B
30 min
CIDU
30 min
IDU
DNA fiber combing
Signal Length
Fork Symmetry
Inter Origin Distance (IOD)
Replication rate (RR)
Fork stability (FS)
C
Replication rate [kb/min]
ns
0.0049
0.0046
< 0.0001
0.0004
D
Inter-Origin Distance [kb]
0.0092
0.0138
E
DNA
γH2Ax
Merge
HCT116
% of cells with γH2Ax foci
0.0411

Appendix Figure S6 - Changes in replication after *in vitro* evolution

A. Extended version of representative immunoblot of MCM2-7 in model cell lines before and after *in vitro* evolution (Figure 3C).

B. Schematic depiction of the replication dynamics analysis.

C. Replication rate of the model cell lines before and after evolution.

D. Inter-origin distance in model aneuploid cells before and after evolution.

E. Representative image and quantification of γH2Ax immunofluorescence before and after *in vitro* evolution in polysomic cells. Three biological replicates; At least 5000 nuclei were scored with each sample. Scale bars in microscopy images: 10 μm.

Data information: Bar plots show mean with SEM. P-values were calculated using unpaired Student's t-test.

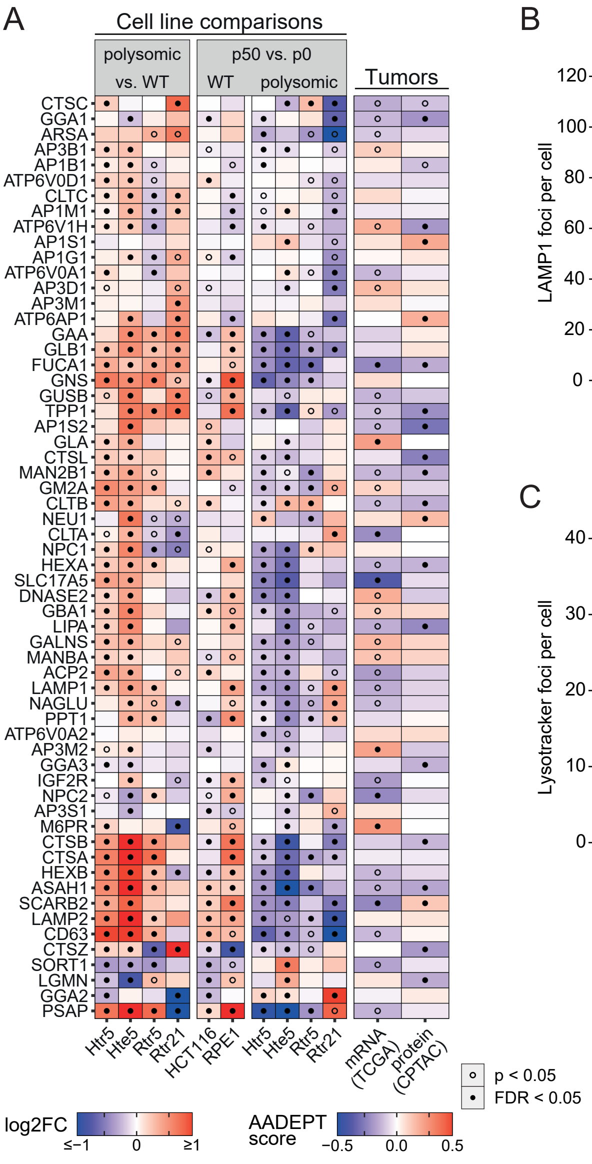
A
Cell line comparisons
polysomic vs. WT
p50 vs. p0
WT
polysomic
Tumors
CTSC
GGA1
ARSA
AP3B1
AP1B1
ATP6V0D1
CLTC
AP1M1
ATP6V1H
AP1S1
AP1G1
ATP6V0A1
AP3D1
AP3M1
ATP6AP1
GAA
GLB1
FUCA1
GNS
GUSB
TPP1
AP1S2
GLA
CTSL
MAN2B1
GM2A
CLTB
NEU1
CLTA
NPC1
HEXA
SLC17A5
DNASE2
GBA1
LIPA
GALNS
MANBA
ACP2
LAMP1
NAGLU
PPT1
ATP6V0A2
AP3M2
GGA3
IGF2R
NPC2
AP3S1
M6PR
CTSB
CTSA
HEXB
ASAH1
SCARB2
LAMP2
CD63
CTSZ
SORT1
LGMN
GGA2
PSAP
Htr5
Hte5
Rtr5
Rtr21
HCT116
RPE1
Htr5
Hte5
Rtr5
Rtr21
mRNA (TCGA)
protein (CPTAC)
p < 0.05
FDR < 0.05
log2FC
≤−1
0
≥1
AADEPT score
−0.5
0.0
0.5
B
<0.0001
<0.0001
<0.0001
LAMP1 foci per cell
0
20
40
60
80
100
120
p0
p50
p0
p50
HCT116
Htr5
<0.0001
<0.0001
LAMP2 foci per cell
0
20
40
60
p0
p50
p0
p50
HCT116
Htr5
C
<0.0001
<0.0001
Lysotracker foci per cell
0
10
20
30
40
p0
p50
p0
p50
HCT116
Htr5

Appendix Figure S7 - Changes in lysosome and lysosome degradation pathways and phenotypes after *in vitro* evolution

A. Expression changes of lysosomal proteins (KEGG) after chromosome gain (polysomic vs. WT) and after *in vitro* evolution (p50 vs. p0) and their corresponding AADEPT scores (as in Figure 2E).

B. Quantification of immunofluorescence intensity of LAMP1 and LAMP2. Scatter plot of foci per cell ($n \geq 3600$, three independent experiments with three technical replicates each). For the LAMP1 analysis values greater than 120 foci per cell were excluded since they were most likely artifacts of high background.

C. Quantification of cells with lysotracker foci. Scatter plot of foci per cell ($n \geq 2400$, three independent experiments with three technical replicates each.

Data information: Error bars represent mean with SEM. P-values were calculated using unpaired Student's t-test.

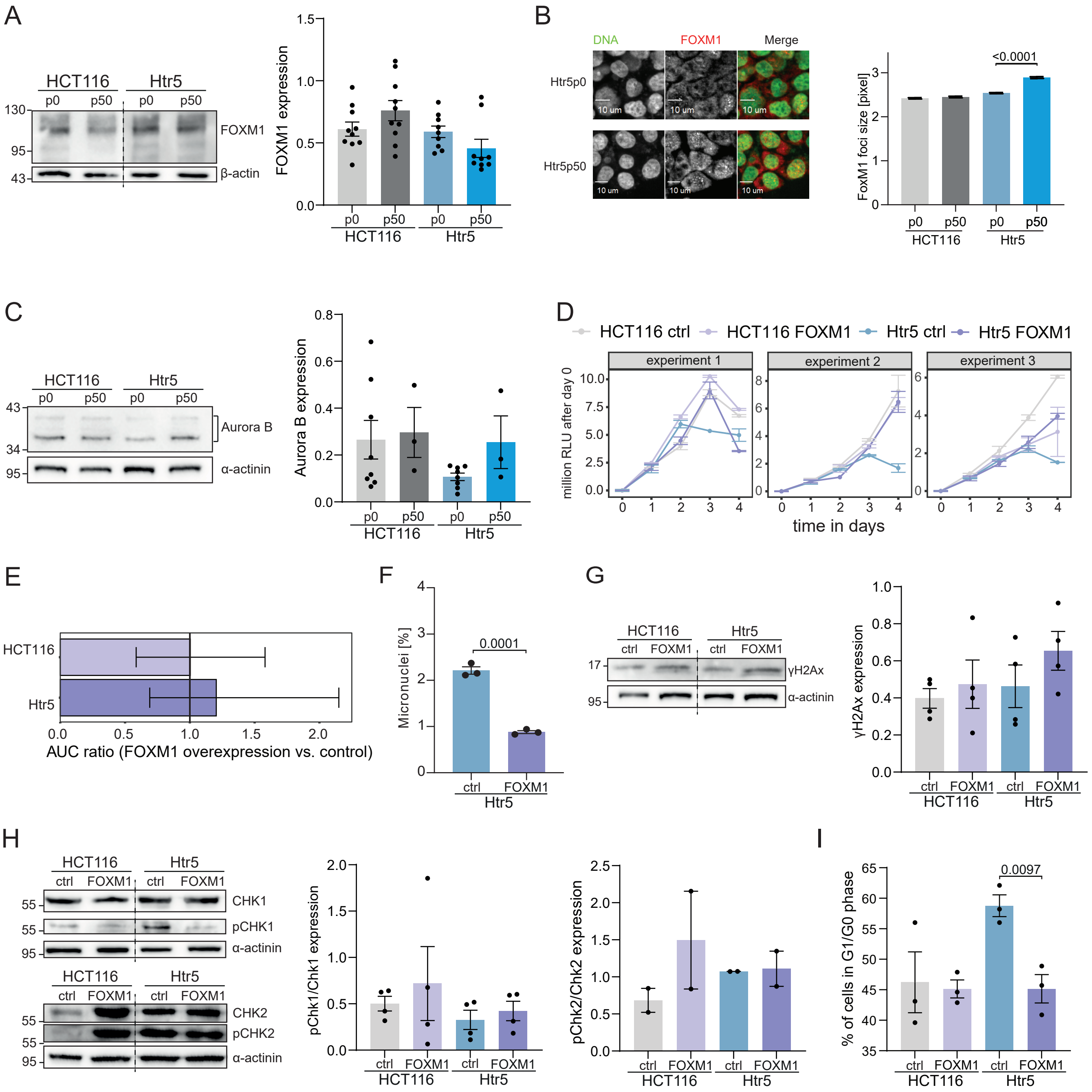
A
HCT116
Htr5
p0
p50
FOXM1
β-actin
FOXM1 expression
B
DNA
FOXM1
Merge
Htr5p0
Htr5p50
FoxM1 foci size [pixel]
<0.0001
C
Aurora B
α-actinin
Aurora B expression
D
HCT116 ctrl
HCT116 FOXM1
Htr5 ctrl
Htr5 FOXM1
experiment 1
experiment 2
experiment 3
million RLU after day 0
time in days
E
AUC ratio (FOXM1 overexpression vs. control)
F
Micronuclei [%]
0.0001
ctrl
G
γH2Ax
γH2Ax expression
H
CHK1
pCHK1
CHK2
pCHK2
pChk1/Chk1 expression
pChk2/Chk2 expression
I
% of cells in G1/G0 phase
0.0097

Appendix Figure S8 - FOXM1 dependent changes after *in vitro* evolution and in cancer

A. Representative immunoblot of FOXM1 expression and quantification before and after evolution (n: 9 - 10).

B. Representative image and quantification of FOXM1 immunofluorescence. At least 15.000 cells per sample were evaluated, three biological replicates, 30 captures each. Scale bars in microscopy images: 10 μm.

C. Representative immunoblot of Aurora B kinase expression and quantification before and after evolution (n: 3 - 8).

D. Growth curves from all MTT assay experiments of population growth of HCT116 and Htr5 with and without FOXM1 over expression. Points represent mean relative light units (RLU) normalized to the time point 0 with SEM.

E. Ratios of AUCs between FOXM1 overexpression and control cell lines averaged over experiments. Bars represent empirical 95% confidence intervals (10.000 bootstrap samples).

F. Quantification of percentage of micronuclei. Three biological replicates; 50 captures per sample.

G. Representative immunoblot of yH2Ax and quantification (n = 3).

H. Representative immunoblots of Chk1, pChk1 and Chk2, pChk2 and quantification. Ratio phosphorylated to total protein expression (n: 2 - 4).

I. The fraction of G1/G0 cells determined by flow cytometry. Mean of three biological replicates with 100.000 cells tested for each.

Data information: Bar plots show mean with SEM. P-values were calculated using unpaired Student's t-test.

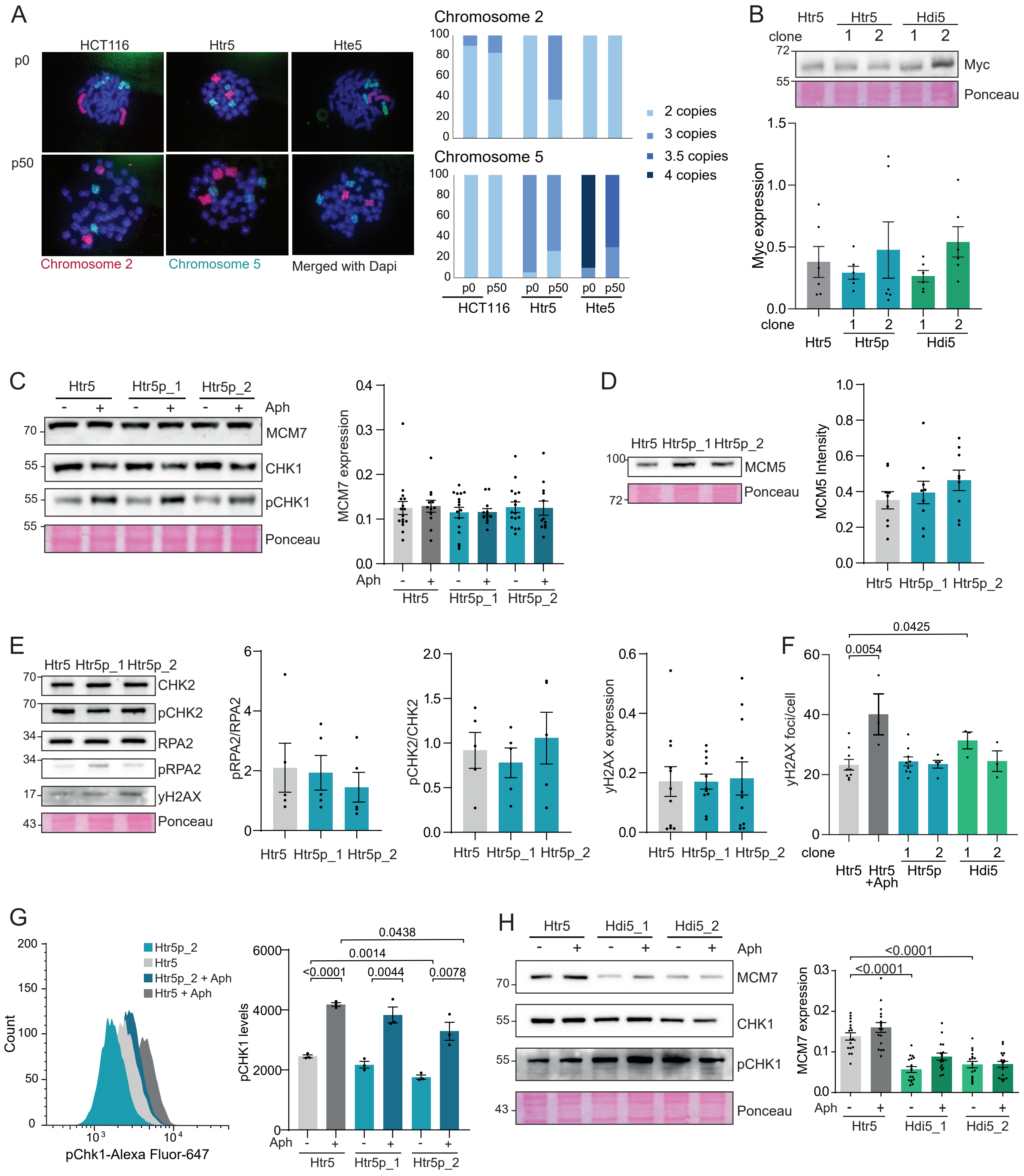
A
HCT116
Htr5
Hte5
p0
p50
Chromosome 2
Chromosome 5
Merged with Dapi
Chromosome 2
Chromosome 5
2 copies
3 copies
3.5 copies
4 copies
p0 p50 p0 p50 p0 p50
HCT116 Htr5 Hte5
B
Htr5 Htr5 Hdi5
clone 1 2 1 2
Myc
Ponceau
Myc expression
clone
Htr5 Htr5p Hdi5
C
Htr5 Htr5p_1 Htr5p_2
Aph
MCM7
CHK1
pCHK1
Ponceau
MCM7 expression
D
Htr5 Htr5p_1 Htr5p_2
MCM5
Ponceau
MCM5 Intensity
E
Htr5 Htr5p_1 Htr5p_2
CHK2
pCHK2
RPA2
pRPA2
yH2AX
Ponceau
pRPA2/RPA2
pCHK2/CHK2
yH2AX expression
F
0.0425
0.0054
yH2AX foci/cell
clone
Htr5 Htr5 +Aph Htr5p Hdi5
G
Htr5p_2
Htr5
Htr5p_2 + Aph
Htr5 + Aph
Count
pChk1-Alexa Fluor-647
0.0438
0.0014
<0.0001
0.0044
0.0078
pCHK1 levels
Aph
Htr5 Htr5p_1 Htr5p_2
H
Htr5 Hdi5_1 Hdi5_2
Aph
MCM7
CHK1
pCHK1
Ponceau
<0.0001
<0.0001
MCM7 expression
Htr5 Hdi5_1 Hdi5_2

Appendix Figure S9 - Effect of chromosome 5 arms on replication stress-related protein expression

A. Quantification of the chromosome arm copy number changes before and after the *in vitro* evolution.

B. Representative immunoblot of MYC expression in Htr5, Htr5p and Hdi5 cell lines with quantification.

C. Representative immunoblot of MCM7 and pCHK1(S345)/CHK1 expression in Htr5 and Htr5p cell lines with MCM7 quantification.

D. Representative immunoblot of MCM5 expression with quantification.

E. Representative immunoblot of CHK2, pCHK2, RPA2, pRPA2 (S33), and yH2AX with respective quantifications.

F. Quantification of yH2AX immunofluorescence.

G. Representative histograms of pCHK1 flow cytometry analysis with quantification.

H. Representative immunoblot of MCM7, CHK1, and pCHK1(S345) expression in Htr5 and Hdi5 cell lines with MCM7 quantification. All experiments were performed in at least three biological replicates with 1 to 6 technical replicates each.

Data information: Bar plots show mean with SEM. P-values were calculated using unpaired Student’s t-test.
