# Supplementary material for: Proteogenomic analysis reveals adaptive strategies for alleviating the consequences of aneuploidy in cancer
Source: EMBO J. 2025 Feb 10;44(6):1829–65. doi: 10.1038/s44318-025-00372-w (PMC11914506; doi:10.1038/s44318-025-00372-w)
Supplement: Supplementary file 15 — Expanded View Figures [file 44318_2025_372_MOESM15_ESM.pdf]

# Expanded View Figures

**Figure EV1. Gene expression changes of individual proteins after in vitro evolution and during tumorigenesis.**

(**A**) Venn diagrams visualizing the overlap in significantly over- and underabundant proteins (FDR < 0.05) for cell line comparisons between polysomic cell lines and the parental WT (p0 vs. WT) and between polysomic cell lines before and after evolution (p50 vs. p0). (**B**) Volcano plots showing changes in relative protein abundance after in vitro evolution of polysomic and WT cell lines ($n = 3$ biological replicates per cell line). Proteins significantly over- (red) and underabundant (blue) in all adapted polysomic cell lines are highlighted next to the top 5 most over- and underabundant proteins per individual comparison. (**C**) Volcano plots of AADEPT scores for all measured genes on transcript- (TCGA) and protein-level (CPTAC) derived from 442 to 467 and 39 to 247 cancer patients respectively. Genes with top 10 lowest and highest AADEPT scores as well as genes of proteins in the model cell line overlap are highlighted.

© The Author(s)

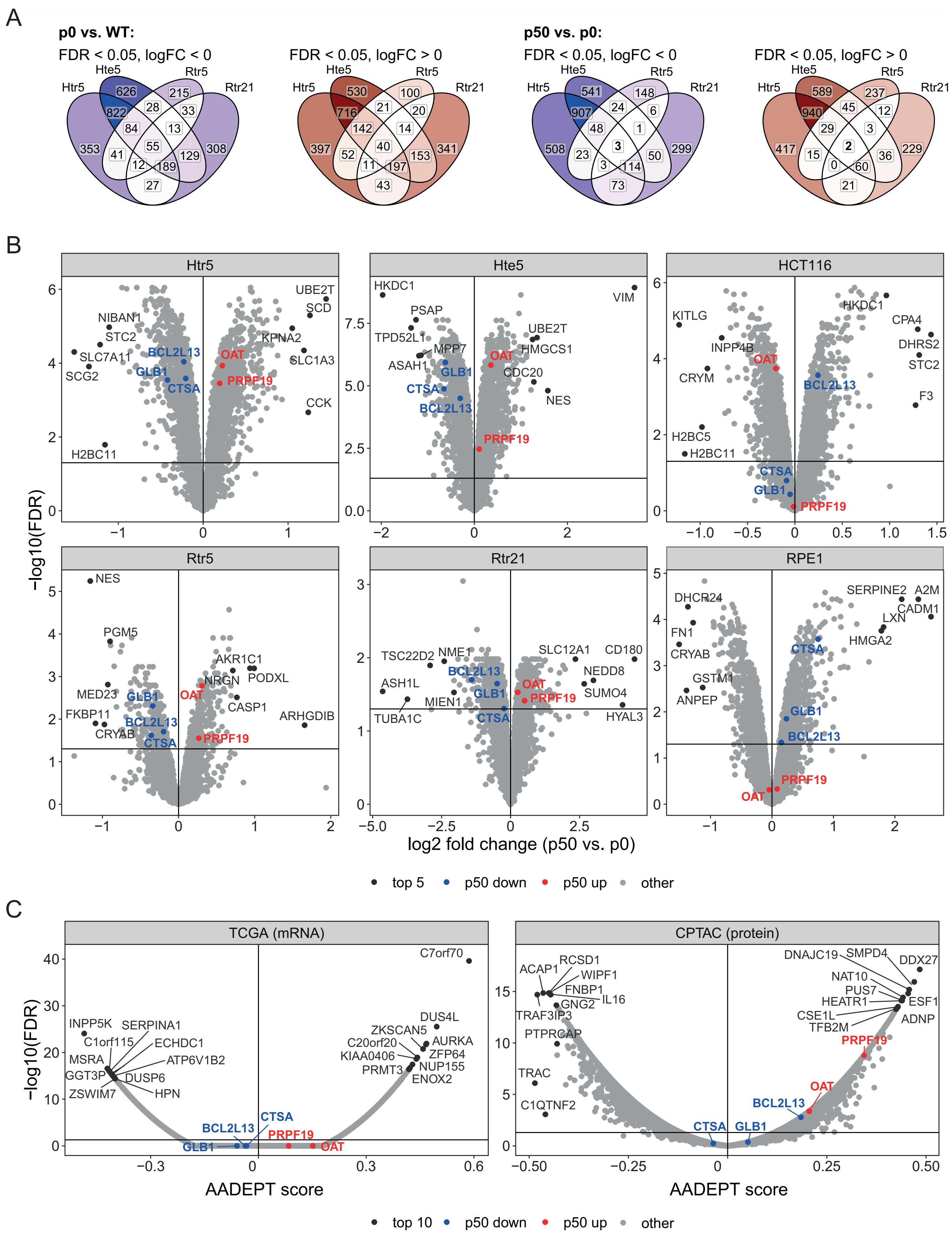

A
p0 vs. WT:
FDR < 0.05, logFC < 0
FDR < 0.05, logFC > 0
p50 vs. p0:
FDR < 0.05, logFC < 0
FDR < 0.05, logFC > 0
Htr5
Hte5
Rtr5
Rtr21
B
Htr5
Hte5
HCT116
Rtr5
Rtr21
RPE1
−log10(FDR)
log2 fold change (p50 vs. p0)
top 5
p50 down
p50 up
other
C
TCGA (mRNA)
CPTAC (protein)
AADEPT score
top 10

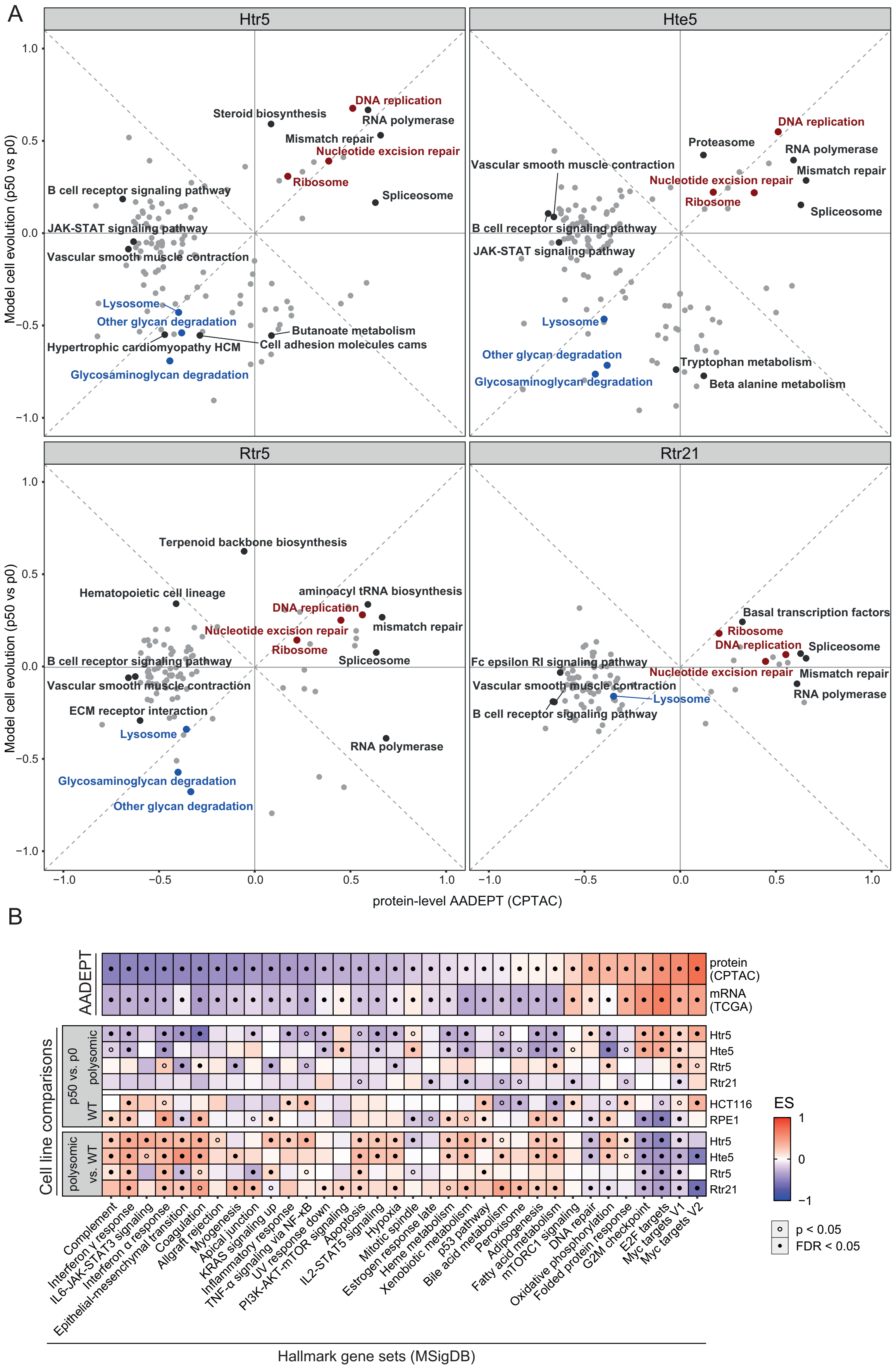
A
Htr5
Hte5
Rtr5
Rtr21
Model cell evolution (p50 vs p0)
protein-level AADEPT (CPTAC)
DNA replication
Steroid biosynthesis
RNA polymerase
Mismatch repair
Nucleotide excision repair
Ribosome
Spliceosome
B cell receptor signaling pathway
JAK-STAT signaling pathway
Vascular smooth muscle contraction
Lysosome
Other glycan degradation
Butanoate metabolism
Hypertrophic cardiomyopathy HCM
Cell adhesion molecules cams
Glycosaminoglycan degradation
Proteasome
Tryptophan metabolism
Beta alanine metabolism
Terpenoid backbone biosynthesis
Hematopoietic cell lineage
aminoacyl tRNA biosynthesis
mismatch repair
ECM receptor interaction
Basal transcription factors
Fc epsilon RI signaling pathway
B
AADEPT
protein (CPTAC)
mRNA (TCGA)
Cell line comparisons
p50 vs. p0
polysomic
WT
polysomic vs. WT
Htr5
Hte5
Rtr5
Rtr21
HCT116
RPE1
ES
1
0
−1
p < 0.05
FDR < 0.05
Complement
Interferon γ response
IL6-JAK-STAT3 signaling
Interferon α response
Epithelial-mesenchymal transition
Coagulation
Allograft rejection
Myogenesis
Apical junction
KRAS signaling up
Inflammatory response
TNF-α signaling via NF-κB
UV response down
PI3K-AKT-mTOR signaling
Apoptosis
IL2-STAT5 signaling
Hypoxia
Mitotic spindle
Estrogen response late
Heme metabolism
Xenobiotic metabolism
p53 pathway
Bile acid metabolism
Peroxisome
Adipogenesis
Fatty acid metabolism
mTORC1 signaling
DNA repair
Oxidative phosphorylation
Folded protein response
G2M checkpoint
E2F targets
Myc targets V1
Myc targets V2
Hallmark gene sets (MSigDB)

◀ **Figure EV2. Gene expression changes of individual pathways after in vitro evolution and during tumorigenesis.**

(**A**) 2D enrichment of KEGG pathways comparing protein-level AADEPT scores (*x* axis) with fold changes in all polysomic cell lines after adaptation (*y* axis), showing enrichment scores for pathways with FDR < 0.05 and highlighting those with shared enrichment in adapted polysomic cell lines (blue—negative, red—positive) as well as those with top 3 enrichment scores in both direction of either axis and with more than 10 measured proteins. (**B**) Enrichment scores of Hallmark gene sets for AADEPT scores (above) and for protein abundance changes between cell line comparisons (below). Hallmark gene sets with FDR < 0.05 in a 2D enrichment analysis of transcript- and protein-level AADEPT scores are shown and sorted by degree of enrichment with protein-level AADEPT scores. The statistical significance of enrichment for individual gene sets was calculated using univariate ANOVA (see "Methods") and is indicated by the points within each tile.

 © The Author(s)

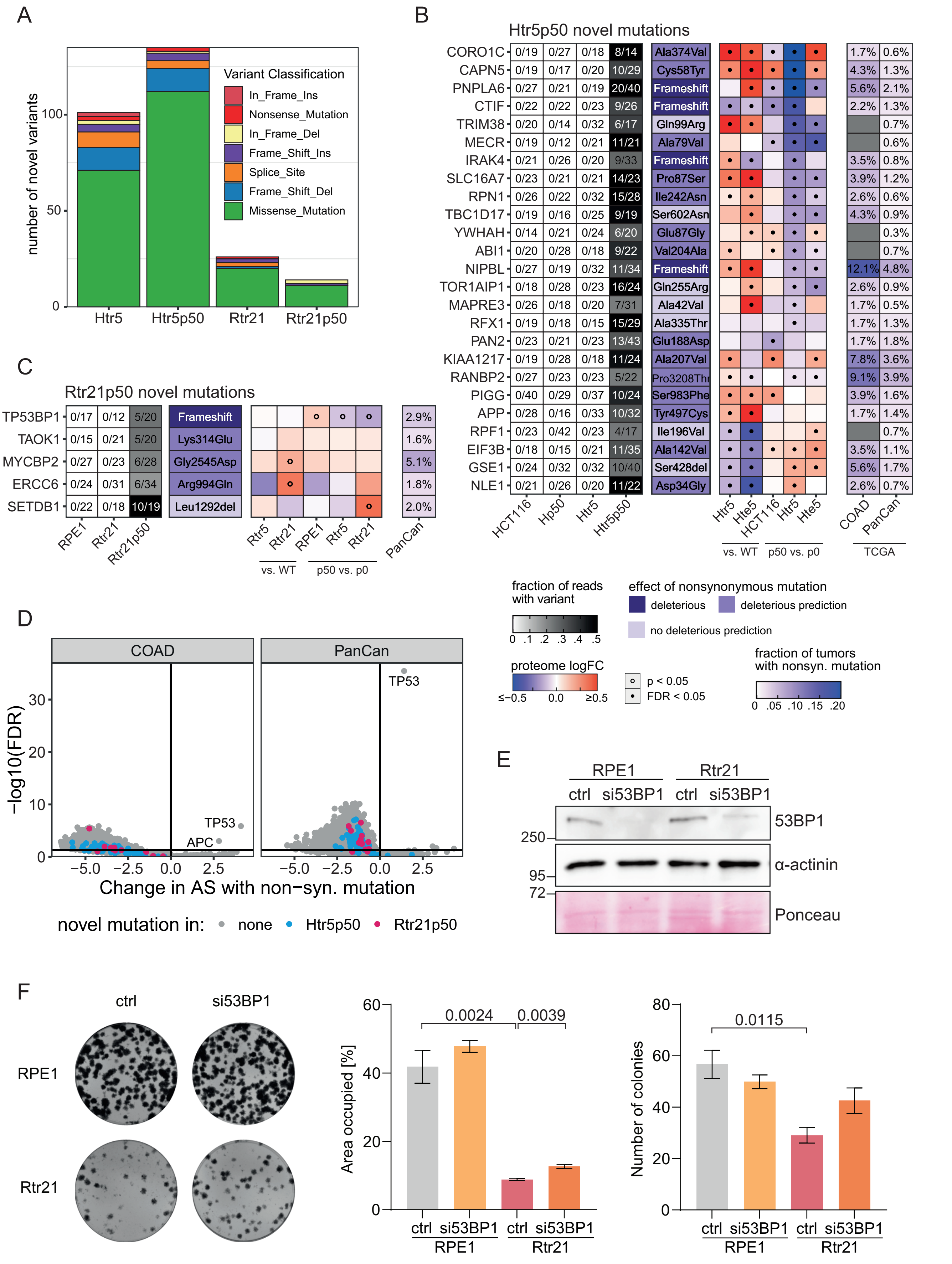
A
number of novel variants
Variant Classification
In_Frame_Ins
Nonsense_Mutation
In_Frame_Del
Frame_Shift_Ins
Splice_Site
Frame_Shift_Del
Missense_Mutation
Htr5
Htr5p50
Rtr21
Rtr21p50
B
Htr5p50 novel mutations
CORO1C 0/19 0/27 0/18 8/14 Ala374Val 1.7% 0.6%
CAPN5 0/19 0/17 0/20 10/29 Cys58Tyr 4.3% 1.3%
PNPLA6 0/27 0/21 0/19 20/40 Frameshift 5.6% 2.1%
CTIF 0/22 0/22 0/23 9/26 Frameshift 2.2% 1.3%
TRIM38 0/20 0/14 0/32 6/17 Gln99Arg 0.7%
MECR 0/19 0/12 0/21 11/21 Ala79Val 0.6%
IRAK4 0/21 0/26 0/20 9/33 Frameshift 3.5% 0.8%
SLC16A7 0/23 0/21 0/21 14/23 Pro87Ser 3.9% 1.2%
RPN1 0/26 0/22 0/32 15/28 Ile242Asn 2.6% 0.6%
TBC1D17 0/19 0/16 0/25 9/19 Ser602Asn 4.3% 0.9%
YWHAH 0/14 0/21 0/24 6/20 Glu87Gly 0.3%
ABI1 0/20 0/28 0/18 9/22 Val204Ala 0.7%
NIPBL 0/27 0/19 0/32 11/34 Frameshift 12.1% 4.8%
TOR1AIP1 0/18 0/28 0/23 16/24 Gln255Arg 2.6% 0.9%
MAPRE3 0/26 0/18 0/20 7/31 Ala42Val 1.7% 0.5%
RFX1 0/19 0/18 0/15 15/29 Ala335Thr 1.7% 1.3%
PAN2 0/23 0/21 0/23 13/43 Glu188Asp 1.7% 1.8%
KIAA1217 0/19 0/28 0/18 11/24 Ala207Val 7.8% 3.6%
RANBP2 0/27 0/23 0/23 5/22 Pro3208Thr 9.1% 3.9%
PIGG 0/40 0/29 0/37 10/24 Ser983Phe 3.9% 1.6%
APP 0/28 0/16 0/33 10/32 Tyr497Cys 1.7% 1.4%
RPF1 0/23 0/42 0/23 4/17 Ile196Val 0.7%
EIF3B 0/18 0/15 0/21 11/35 Ala142Val 3.5% 1.1%
GSE1 0/24 0/32 0/32 10/40 Ser428del 5.6% 1.7%
NLE1 0/21 0/26 0/20 11/22 Asp34Gly 2.6% 0.7%
HCT116 Hp50 Htr5 Htr5p50
Htr5 Hte5 HCT116 Htr5 Hte5
vs. WT
p50 vs. p0
COAD PanCan
TCGA
C
Rtr21p50 novel mutations
TP53BP1 0/17 0/12 5/20 Frameshift 2.9%
TAOK1 0/15 0/21 5/20 Lys314Glu 1.6%
MYCBP2 0/27 0/23 6/28 Gly2545Asp 5.1%
ERCC6 0/24 0/31 6/34 Arg994Gln 1.8%
SETDB1 0/22 0/18 10/19 Leu1292del 2.0%
RPE1 Rtr21 Rtr21p50
Rtr5 Rtr21 RPE1 Rtr5 Rtr21
vs. WT
p50 vs. p0
PanCan
fraction of reads with variant
0 .1 .2 .3 .4 .5
effect of nonsynonymous mutation
deleterious
deleterious prediction
no deleterious prediction
proteome logFC
≤−0.5 0.0 ≥0.5
p < 0.05
FDR < 0.05
fraction of tumors with nonsyn. mutation
0 .05 .10 .15 .20
D
COAD
PanCan
−log10(FDR)
TP53
APC
Change in AS with non−syn. mutation
novel mutation in: none Htr5p50 Rtr21p50
E
RPE1
Rtr21
ctrl si53BP1 ctrl si53BP1
250
95
72
53BP1
α-actinin
Ponceau
F
ctrl
si53BP1
RPE1
Rtr21
Area occupied [%]
0.0024
0.0039
Number of colonies
0.0115
ctrl si53BP1 ctrl si53BP1
RPE1
Rtr21

**Figure EV3. Novel point mutations after in vitro evolution.**

(**A**) Number of variants with classification per cell lines, excluding parental variants or those observed during evolution of diploid HCT116. (**B**) Fraction of variant reads per cell line for genes exclusively mutated in evolved Htr5 together with combined SIFT and PolyPhen variant effect prediction, changes in protein abundance, and prevalence of nonsynonymous mutations in TCGA colon (COAD) and pan-cancer tumors. Statistical significance for protein abundance changes between cell lines was determined using empirical Bayes moderated Student's *t* tests (see "Methods"). (**C**) Same as (**B**), but for genes exclusively mutated in evolved Rtr21. Legends, including numbered scale bars for heatmaps, are the same as in (**B**). (**D**) Volcano plot with size and significance of difference in average aneuploidy score between tumors with and without nonsynonymous mutations in a respective gene in colon and pan-cancer tumors. Only genes mutated in at least four samples were considered. *P* values were derived using Wilcoxon rank sum test of aneuploidy scores between the groups. (**E**) Representative immunoblot of 53BP1 in the RPE1 and unevolved Rtr21 48 h after transfection with either siRNA against 53BP1 or scrambled control siRNA. (**F**) Representative images of clonogenic assay of RPE1 and Rtr21 after transfection with either siRNA against 53BP1 or scrambled control siRNA. Quantification of the percentage of area covered by cells and the number of colonies in the clonogenic assay is shown ($n = 1$ for control cells, $n = 3$ for depleted cells, three technical replicates each), mean with SEM is shown. *P* values were calculated using unpaired Student's *t* test.


© The Author(s)

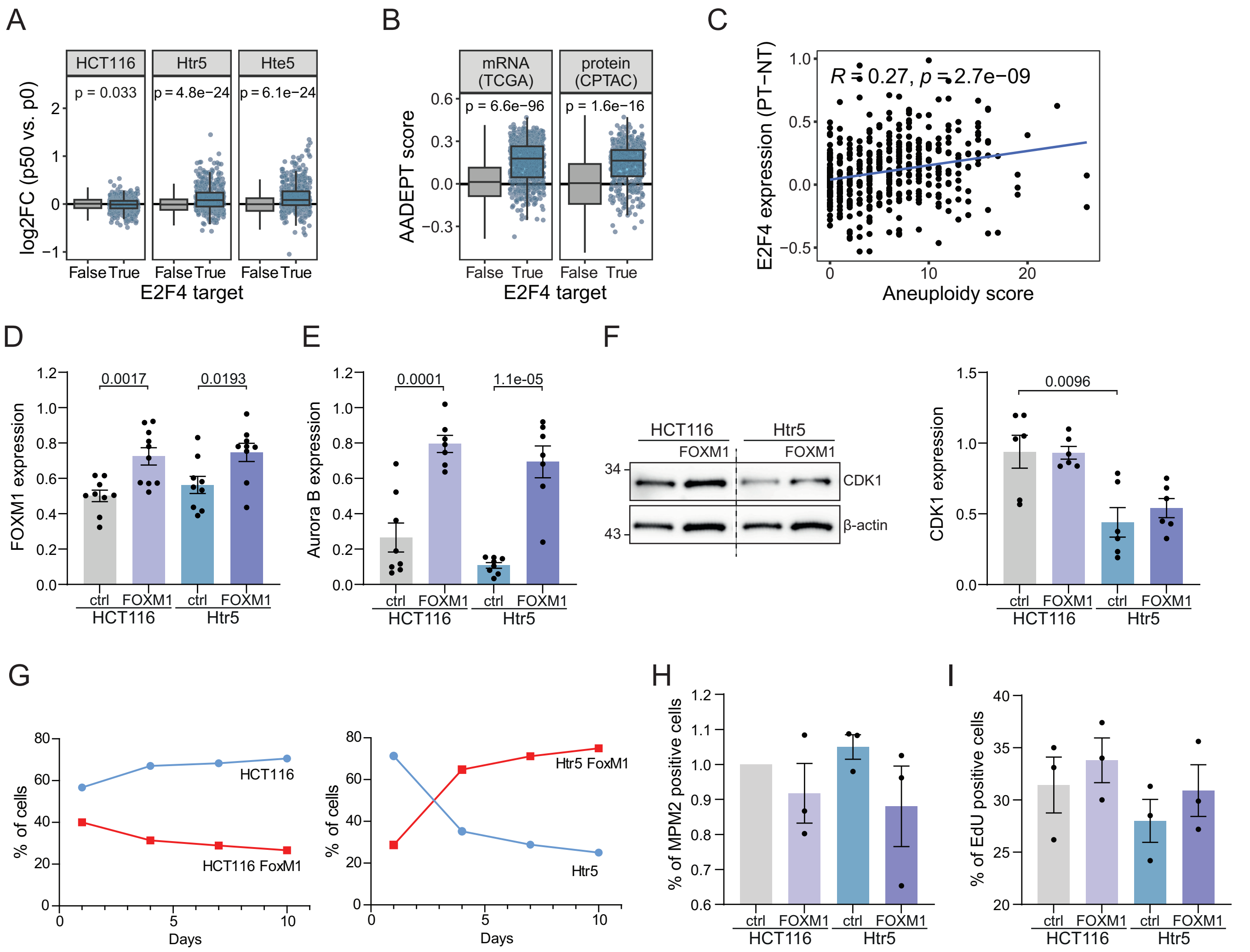


**Figure EV4. FOXM1 and E2F4 dependent changes after in vitro evolution and in cancer.**

(**A**) Protein abundance fold changes of all E2F4 targets ($n = 484$) in evolved model cell lines, tested against all other proteins using Welch's *t* tests. (**B**) Transcript- (TCGA) and protein-level (CPTAC) AADEPT scores of E2F4 target genes ($n = 648$, $n = 406$ respectively) tested against all other proteins using Welch's *t* tests. (**C**) Spearman correlation coefficient between TCGA patient ($N = 467$) tumor aneuploidy score and E2F4 gene expression relative to normal tissue. (**D**) Quantification of immunoblotting of FOXM1 overexpression cells of three biological replicates with 3 to 4 technical replicates each. (**E**) Quantification of immunoblotting of Aurora kinase B in FOXM1 overexpressing cells of three biological replicates with 2 to 3 technical replicates. (**F**) Representative immunoblot of CDK1 in FOXM1 overexpressing cells and quantification of three biological replicates with two technical replicates each. (**G**) Quantification of the RFP- and BFP-positive cell fraction in competition assay. Representative experiment. (**H**) The fraction of phospho-MPM-2 positive cells determined by flow cytometry. Mean of 3 biological replicates with 100,000 cells tested for each. (**I**) The fraction of EdU-positive cells determined by flow cytometry. Mean of 3 biological replicates with 100,000 cells tested for each. Data information: Boxplots represent the 25th and 75th percentile with the median. The whiskers extend from the upper and lower bound of the box to the largest and smallest value no further than 1.5 * IQR (inter-quartile range) from the respective bound (Tukey method). Bar plots show mean with SEM. If not specified otherwise, *p* values were calculated using unpaired Student's *t* test.

© The Author(s)

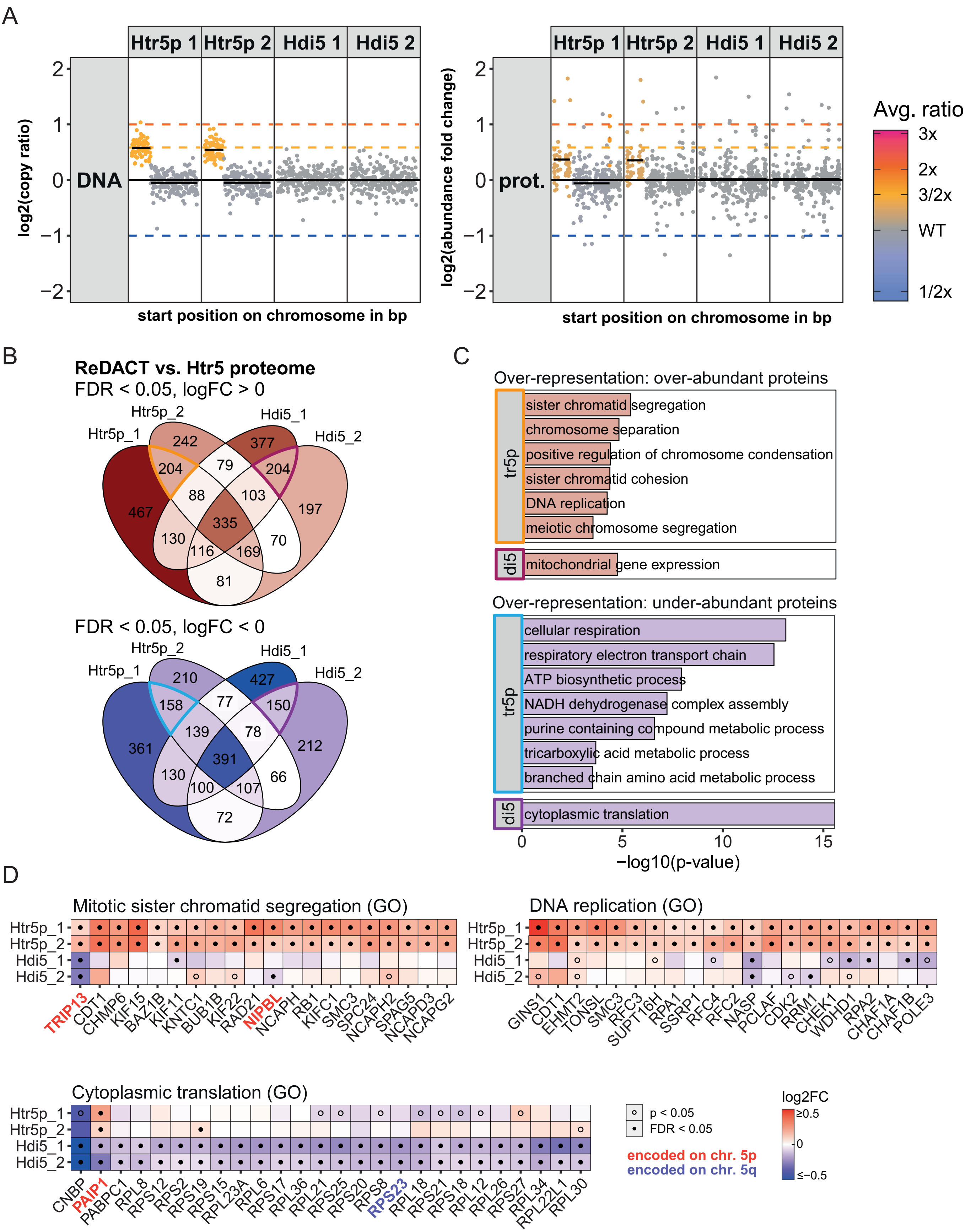
A
Htr5p 1
Htr5p 2
Hdi5 1
Hdi5 2
DNA
prot.
log2(copy ratio)
log2(abundance fold change)
start position on chromosome in bp
Avg. ratio
3x
2x
3/2x
WT
1/2x
B
ReDACT vs. Htr5 proteome
FDR < 0.05, logFC > 0
FDR < 0.05, logFC < 0
Htr5p_1
Htr5p_2
Hdi5_1
Hdi5_2
C
Over-representation: over-abundant proteins
sister chromatid segregation
chromosome separation
positive regulation of chromosome condensation
sister chromatid cohesion
DNA replication
meiotic chromosome segregation
mitochondrial gene expression
Over-representation: under-abundant proteins
cellular respiration
respiratory electron transport chain
ATP biosynthetic process
NADH dehydrogenase complex assembly
purine containing compound metabolic process
tricarboxylic acid metabolic process
branched chain amino acid metabolic process
cytoplasmic translation
tr5p
di5
−log10(p-value)
D
Mitotic sister chromatid segregation (GO)
DNA replication (GO)
Cytoplasmic translation (GO)
p < 0.05
FDR < 0.05
encoded on chr. 5p
encoded on chr. 5q
log2FC
≥0.5
≤−0.5

 © The Author(s)

**Figure EV5. Differential protein abundance in trisomy 5 ReDACT clones.**

(**A**) Average DNA copy number ratios of genomic bins (left) and abundance fold changes of proteins (right) relative to disomic HCT116. Only genomic bins and proteins located or encoded on chromosome 5 are shown for Htr5p and Hdi5 ReDACT clones in order of genomic start position. (**B**) Overlap of over- (top) and underabundant (bottom) proteins compared to parental Htr5 between Htr5p and Hdi5 clones. Proteins exclusive to Htr5p and Hdi5 are highlighted respectively. (**C**) Biological processes (Gene Ontology Consortium et al, 2023, Data ref: Liberzon et al, 2023) over-represented by the sets of proteins highlighted in (**A**). The redundancy of results was reduced using affinity propagation (see "Methods"). (**D**) Cell line-wise abundance fold changes of proteins which are involved in selected biological processes from the results of (**B**) and which are part of the respective set of exclusively deregulated proteins of (**A**). Statistical significance for protein abundance changes between cell lines was determined using empirical Bayes moderated Student's *t* tests (see "Methods").

© The Author(s)
